# Population size and structure of beisa oryx and gerenuk in Geralle National Park, southeastern Ethiopia

**DOI:** 10.1002/ece3.70008

**Published:** 2024-07-15

**Authors:** Melkamu Aychew, Zerihun Girma

**Affiliations:** ^1^ Geralle National Park, Ethiopian Wildlife Conservation Authority Addis Ababa Ethiopia; ^2^ Department of Wildlife & Ecotourism Management Wondo Genet College of Forestry & Natural Resource Hawassa University Shshemene Ethiopia

**Keywords:** abundance, density, distance sampling, habitat, season, species

## Abstract

The study was conducted to determine the population size of endangered *Oryx beisa* (Rüppell, 1835), and near‐threatened *Litocranius walleri* (Brooke, 1878) of uncertain global population estimates in Geralle National Park, southeastern Ethiopia. Systematic line transects were established with a transect length range of 2.3 to 6.8 km long (a total of 165.4 km long with a sighting distance of 150 m after truncation). The combination of AIC and chi‐square *p*‐values was used as model selection criteria for density/population size estimation in distance sampling software. The lowest AIC, ∆AIC (close to zero), and Chi‐square tests (*p*‐value > .05) were selected with adequate model fit. The minimum observation was 67 individuals of beisa oryx in the dry season. The maximum observation was 349 individuals of gerenuk during the wet season. The minimum detection probability of oryx was in the wet season (*p*_â_ = 76 ± 26), and the minimum detection probability of gerenuk was (*p*_â_ = 75 ± 1) in both seasons. The two‐season pooled density analyzed for studied species indicated (0.85 ± 0.34, 1.24 ± 0.47, beisa oryx/km^2^), and (3.82 ± 0.6, 4.88 ± 0.7 gerenuk/km^2^) in dry and wet seasons, respectively. It can be concluded from the results of the study that GNP is home to previously undiscovered healthy populations of the endangered beisa oryx and near‐threatened gerenuk. So it is recommended to undergo in‐depth population studies, including other species available in the national park and their habitat components, so as to design sound, sustainable conservation measures for the wildlife resources in the area.

## Graphical abstract

A study was conducted to estimate populations of one endangered (*Oryx beisa*) and near threatened (*Litocranius walleri*) that their global population estimate is very uncertain. The two‐season pooled density analyzed for studied species indicated (0.85 ± 0.34, 1.24 ± 0.47, beisa oryx/km^2^) and (3.82 ± 0.6, 4.88 ± 0.7 gerenuk/km^2^) in dry and wet seasons, respectively. It can be concluded from the results of the study that GNP is home to previously undiscovered viable populations of the endangered beisa oryx and near‐threatened gerenuk.

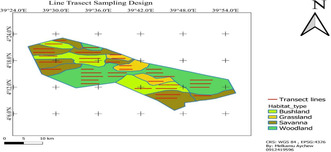

## 1 INTRODUCTION

Large herbivores are considered keystone species or ecosystem engineers; they play an important role in regulating vegetation dynamics, nitrogen cycling, and food webs (Frank et al., 2018; Ramsay et al., 2022). However, tropical ungulates have been threatened in most of their ranges, and this has resulted in consequences of ecosystem functioning imbalances, such as climate change (Asefa et al., 2017; Ramsay et al., 2022).

Beisa oryx [*Oryx beisa* (Linn. 1758)] is a large antelope with a compact, muscular body, long, patterned ears, and a stout neck. Their horns are long, slender, and nearly parallel, with ridges on the lower part. Gerenuk (*Litocranius walleri*), whose name means “giraffe‐necked,” is an exceptionally long‐necked antelope with larger ears and eyes on a relatively small head (Kingdon, 1997). In beisa oryx, both sexes have horns that can reach 75–120 cm in length, whereas in gerenuk only males have horns (Kingdon, 1997). Beisa oryx was formerly thought to be a subspecies of the gemsbok (Oryx gazelle); however, Groves and Grubb (2011) reclassified it as a separate species based on morphological and genetic analysis. Two subspecies of gerenuk have been recognized, and they are sometimes considered distinct species: *Litocranus waleri sclateri* (Northern gerenuk) known as *Sclater's gazelle*.

Beisa oryx was historically found in arid savanna, open scrub, and semi‐desert areas of northern Kenya, southeast Somalia, Ethiopia, Eritrea, Sudan, Djibouti, and Uganda (Kingdon, 1997). The gerenuk range stretches from northwest Somalia (Berbera District) west to the Egyptian border and Djibouti. It occurs in dry thorn shrub habitats and deserts in the Horn of Africa (Okello et al., 2016). It is found throughout the Horn of Africa, including southern Djibouti, Somalia, Ethiopia, Kenya, and northeastern Tanzania. In the 1990s, a survey found at least 25,000 beisa oryx across its range, with Kenya accounting for almost two‐thirds of the total population (IUCN, 2024). However, the species' current global population estimate has dropped to 8000 to 9000 individuals, and it is now designated an endangered species by the IUCN red list (IUCN, 2024). The current global population estimate of gerenuk is not certain. However, East (1999) estimated the population before two and a half decades to be 25 individuals and with aerial counting bias correction, the population estimate was up to 95,000 individuals. According to East (1999), the largest populations known occur in southwestern Ethiopia and the northern and eastern rangelands of Kenya. The current population trend of *L*. *waleri* is declining and is considered near threatened on the IUCN red list (Bärmann et al., 2021; IUCN, 2024). There are no known population estimates of the species in Ethiopia.

Beisa oryx used to be found throughout Ethiopia's northeastern, eastern, and southern lowlands, from the Awash Valley northward to the Danakil region, as well as in the lower Rift Valley from Omo north and west, including Mago and Omo National Parks, Murule and Borena controlled hunting areas, and Geralle National Park (Enawgaw, 2004; IUCN, 2024). Populations of the gerenuk have been documented in protected areas such as Awash, Omo, Mago‐Murule‐Chew Bahir, Ogaden, and Geralle National Park (GNP) in Ethiopia (Enawgaw, 2004; Thouless, 1995). There are no updated population estimates of both beisa oryx and gerenuk in Ethiopia. However, some previous rough estimates have been reported for beisa oryx. The population of beisa oryx before three decades was estimated to be between 4000 and 5000 (East, 1997). However, the population of beisa oryx in Ethiopia is reported to be declining steadily. Particularly, research in Awash National Park, located in the main rift valley region, reported serious population decline over four decades, declining from 4020 individuals in 1969 (Robertson, 1970) to ~450 in 2004 (Enawgaw, 2004). More recently, a study at Alledeghi Wildlife Reserve, northeastern Ethiopia, estimated the population of beisa oryx at the locality to be nearly 1120 (Admasu et al., 2016).

The global population estimate of both *Oryx beisa* and *Litocranius waller* is not certain, due to a lack of local population estimates over their ranges. Although there are limited studies on populations of these two species in Ethiopia (Admasu et al., 2016), the exact population estimates in most localities are not known due to a lack of population studies over most of the species localities. Those few population estimates available are outdated, and the current population status of the species could be far below those decades‐old estimates, due to the fact that most of their habitats are under immense continued anthropogenic pressure.

GNP is one of the sites where a healthy population of the species occurs at present and is threatened mainly due to anthropogenic activities (Asefa et al., 2017). The park is under pressure from increased habitat modification due to the local community pastoral lifestyle, which leads to a rapid expansion of human settlements and overgrazing (Asefa et al., 2017). As a result, there is a need for estimation of the species population at the locality so as to propose sound management measures for the management of the species and their habitats. Furthermore, the population estimate at the locality is a step toward a more reliable population estimate of the species in Ethiopia and consequently, for the global population estimate. To this end, the present study attempts to answer research questions such as: What is the exact population size of *Oryx beisa* and *Litocranius waller* at GNP? What can be inferred from the population structure of the two species about the future population prospects of these two species? Therefore, the study is aimed at determining the population size and structure of beisa oryx and gerenuk in GNP.

## 2 MATERIALS AND METHODS

### 2.1 The study area

**FIGURE 1:**
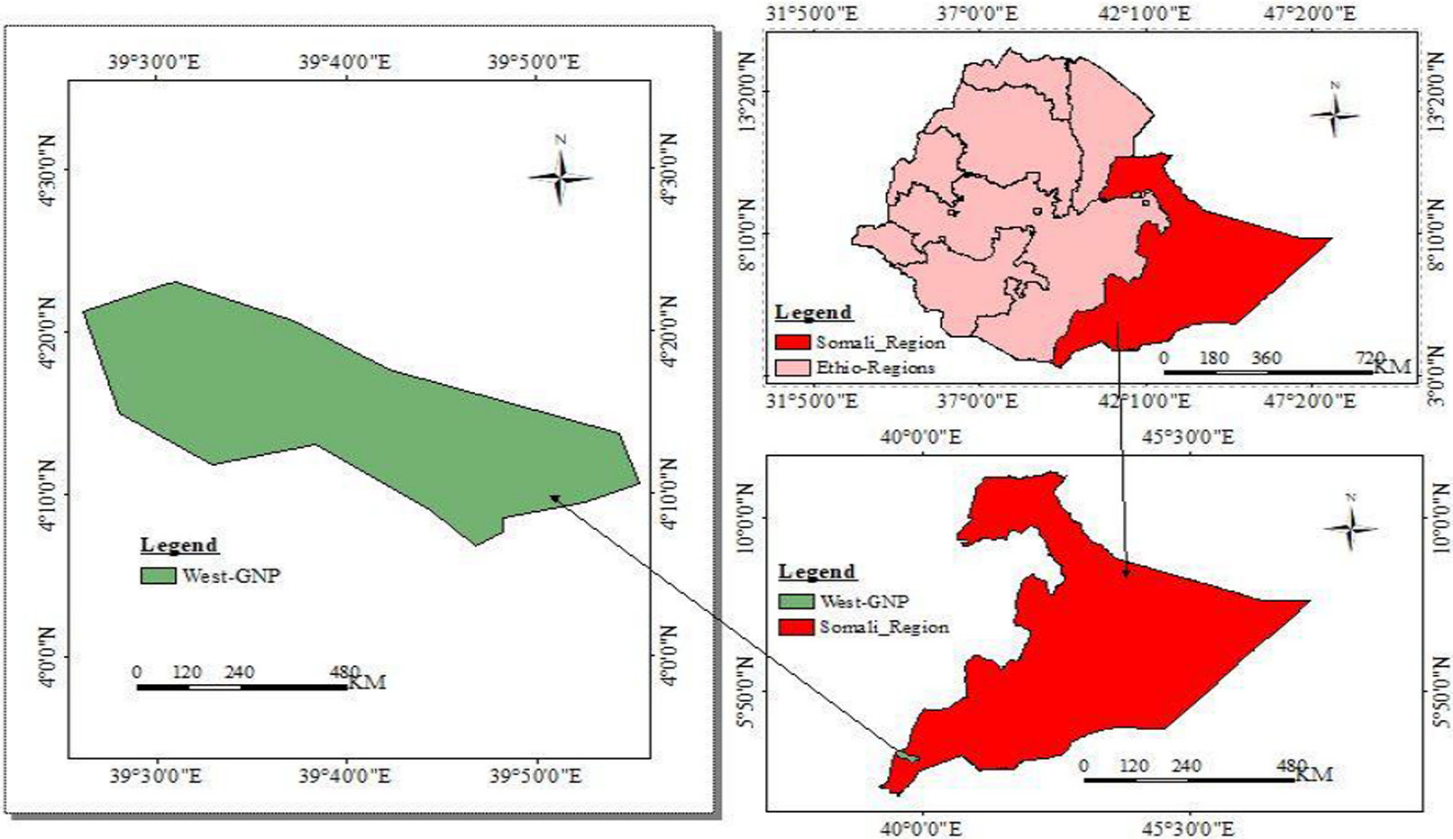
Location map of the study area, West Geralle National Park southeastern Ethiopia (Aychew, 2019).

Geralle National Park (GNP) is located in the southeastern part of Ethiopia in the Somali National Regional State (SNRS) in Dawa Zone, Hudet woreda (Figure 1). The park is divided into west and east parts; the west part covers around 692 km^2^ and the east part covers 1042 km^2^ of the national park, including the adjacent area of the Dawa ecosystem (Asefa et al., 2017). The present study was conducted in the western part of the GNP. West GNP is situated between 4°0′0″ to 4°30′0″ N latitude and 39°30′ to 39° to 39°50′ E long (Figure 1), and its total area coverage is 1734 km^2^. The topography of the National Park is mostly characterized by low land and plains. Altitude ranges as low as 800 m asl on the banks of the Dawa River and as high as 1380 m asl on top of the escarpments (National Meteorological Agency, 2021). The rainfall regime in GNP is bimodal, with a long rainy season between March and May with a peak in April, and a short rainy season between September and November, with a peak in October (National Meteorological Agency, 2021). The mean annual rainfall is 437.31 mm/year (National Meteorological Agency, 2021). The mean annual temperature is 22.97°C. The mean annual maximum temperature is 30.47°C. The mean annual minimum temperature is 15.52°C (Fick & Hijmans, 2017; National Meteorological Agency, 2021).

Geralle National Park is one of the most important wildlife areas in Ethiopia. The park was established in 2006 and is one of the youngest protected areas (PAs) in Ethiopia, with diverse wildlife species. GNP harbors 42 mammalian species and 200 species of birds (Asefa et al., 2017). The main purpose of the establishment of the national park is to conserve some of the remnant threatened and ear threatened species occurring in Geralle National Park. The area is also an important grazing land for the Kenyan and Ethiopian Somali pastoral communities (Asefa et al., 2017).

### 2.2 METHODS

#### 2.2.1 Reconnaissance survey

A reconnaissance survey was conducted to collect basic information on accessibility, topography, and habitat types in March 2019, a week before the actual data collection period. The reconnaissance survey was used for determining; habitat stratification, transect length, width and orientation, and sample size.

#### 2.2.2 Sampling design

A research design was established based on the reconnaissance survey. The study area (Geralle National Park) was stratified into four major habitat types: grassland, bushland, woodland, and wooded grassland, based on dominant vegetation types. Based on the preliminary survey, the total transects length (*L*) needed to estimate abundance with a prescribed CV was estimated using the following equation (Buckland et al., 1993; Thomas et al., 2010):

(1) $$\frac{L_{0}}{n_{0}}\left(\frac{b}{{\mathrm{cv}}_{t}{\left(D\right)}^{2}}\right)$$

where CV_D_ is the desired coefficient of variation for the estimate of density (95% of confidence value which is 0.05), *L*_0_ is the total line length for the pilot study, n_0_ is the number of individuals seen while sampling *L*_0_, and *b* is an estimation of the variability of the number of individuals observed and the probability density function along the line (Buckland et al., 2001). Although difficult to estimate empirically, Burnham et al. (1980) recommend *b* = 3 for planning distance sampling projects.

**FIGURE 2:**
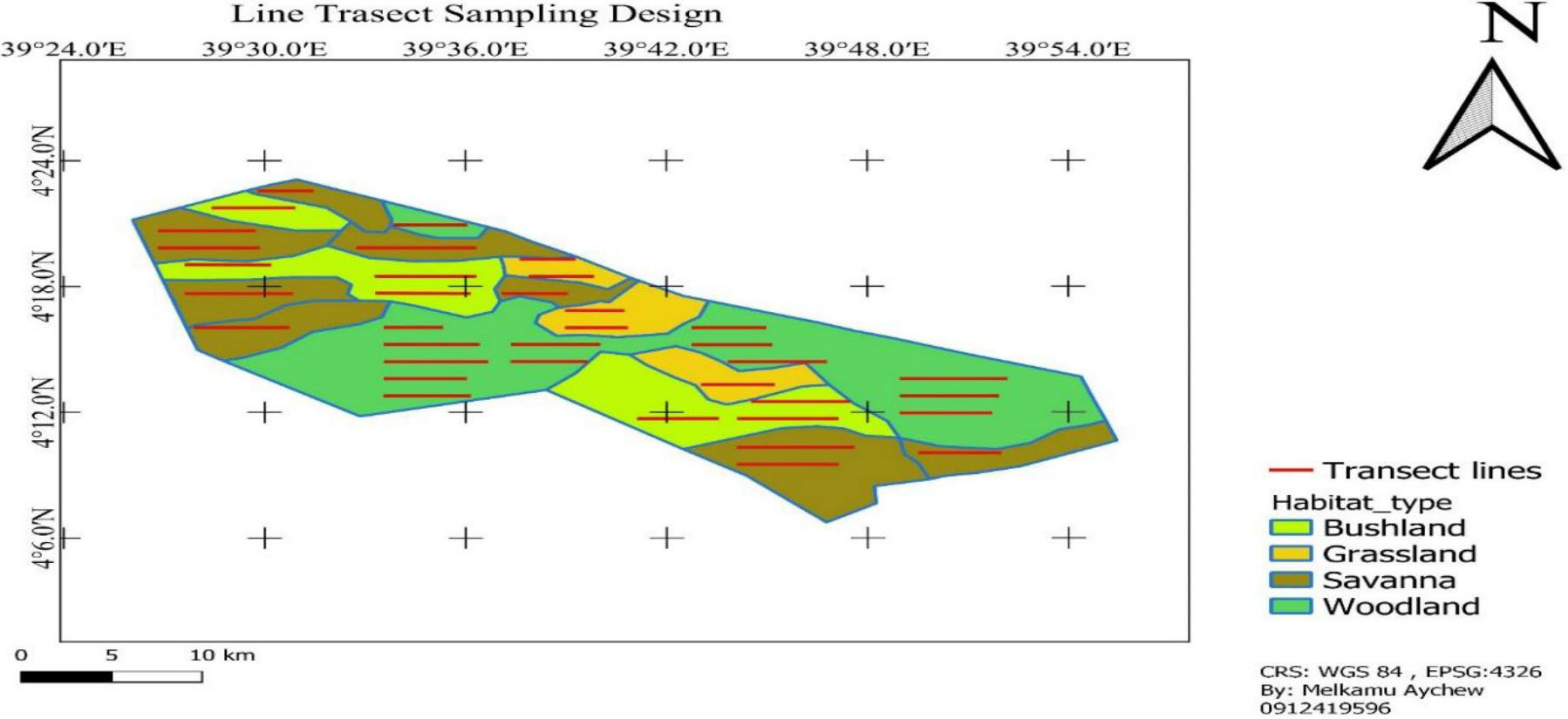
Sampling line transect layout (Aychew, 2019).

Using the formula above, a total of 36 transect lines: five in grassland, seven in bushland, 10 in wooded grassland, and 14 in woodland were established. Transect lines were placed on the stratified habitat types in a systematic random sampling design proportional to the size of the habitat types to minimize sampling bias and achieve representativeness. The transect line varied in length from 2.3 to 6.8 km (Figure 2). The adjacent transects were placed a minimum of 1500 m apart based on vegetation type, transect lines were roughly parallel to each other to avoid double counting, and their ends were at least 5.00 m away from the habitat edges. QGIS was used to plot selected transects using systematic random sampling onto the study area map (Figure 2). The same transects were used for surveys that were conducted during the wet and dry seasons. The marking of the transect line depended on natural markers such as signs of colonies, roads, rocks, and big trees. This method enabled an all‐season survey that was uniform across all habitats.

#### 2.2.3 Data collection

Data were collected during both wet and dry seasons and twice in each season to have enough replicates. Data collection sessions I and II were conducted during the wet season, and sessions III and IV during the dry season. Wet season data collection was carried out from April to May 2019, while dry season data collection was conducted from August to September 2019. Surveys were done during the early morning hours (06:30 to 10:30 a.m.) and late afternoon hours (03:00 to 6:30 p.m.), when most mammals are thought to be active in the study area. Each line transect was navigated by using a global positioning system (GPS) and a handheld bearing compass while walking at a constant speed of ~1 km/h (Peres, 1999). The data collection was made through direct observations with our naked eye and/or by using binoculars. Motorbikes were used in this survey by riding slowly at an average speed of 6 km per hour (Gaidet et al., 2003), motor bicycles were used to survey most of the transect, and walking on foot was only applied in a few places not accessible by motor bicycles. The lowest possible speed for motorbikes was attended so as to standardize the probability of detection with observations obtained on foot walking. During the motorbike survey, maximum care was taken not to disturb animals, employing silent detection methods such as driving at lowest possible speed, wearing camouflaging clothes, and avoiding noises. Local motorbike operators who have a better educational background were carefully selected and trained for 2 days. As observer, scouts with a minimum of 7 years of experience or wildlife expert degree holders in wildlife and related disciplines were selected and trained for 2 days on the data collection techniques. There were a total of three individuals operating motorbikes (three motorbikes involved in data collection), the motorbike operator operated only on the motorbike, and there was one observer for each motorbike. Binoculars were used for proper sex and age identification. Each individual of the two species encountered on both the right and left sides of the transects had data on the number of individuals, sex, and rough age estimate (young, sub‐adult, and adult) recorded. The sighting distance *r* and sighting angle ѳ from the transect line to the animal spotted were measured, and the perpendicular distance was calculated as *r* sin ѳ. Although variable transect width method was employed, perpendicular distance observations that exceed 150 m were discarded for efficiency purpose. The distribution of the observed distances of the animals was used to estimate a “detection function” that describes the probability of detecting an individual animal at a given distance.

Care was taken to minimize the risk of double counting by noticing the movement of mammals between the effective counting widths of adjacent transects. Double counting of the same individual herds was minimized using easily recognizable features of individual herd size (total numbers in the herd), age, distinct body deformations, cut tail and ear, and composition (Wilson & Delahay, 2001).

#### 2.2.4 Data analysis

The data were organized into a data frame, encompassing variables such as stratum, area, perpendicular distance, transect length, cluster size, species, session, and season. The data manipulation and cleaning were performed using R software version 4.3.3. For the analysis of population size, density, and model selection procedures, the distance sampling software version 7.5 (Thomas et al., 2010) supplemented by the MRDS package in R software 4.3.3 was used (Burt et al., 2014; Laake et al., 2020). Variance estimation was grounded on the principles outlined by Buckland et al. (2001) and Fewester et al. (2009) employing the delta method with empirical variance. Each habitat type was analyzed as a separate stratum, and each species and season were analyzed using a data filtering method in a distance software analysis window (Thomas et al., 2010). Population density for each species was calculated as follows:

(2) $$\hat{D}=\frac{n}{2\mathrm{wL}{\hat{p}}_{a}}$$

(Buckland et al., 1993, 2001) where ${\hat{p}}_{a}$ = probability of detection, *n* number of observations, *w* = width of the transect, and *L* = transect distance. Conventional Distance Sampling (CDS) is a widely used methodology for estimating the density or abundance of a population. The MRDS (Multiple‐observer Distance Sampling) analysis engine, which operates on a single observer model, was utilized with a constant parameter from the CDS engine. Despite the MRDS single observer model not introducing additional features compared to the CDS engine, it generates comprehensive summary estimates at both the global and stratum levels when a set of data records is used. These estimates are considered more insightful and detailed than those produced by the CDS engine (Thomas et al., 2010). Both grouped and ungrouped perpendicular distances were performed during model selection procedures to consider categorical and non‐categorical data. The distance model selection procedure was between half‐normal and hazard‐rate key functions for grouped and ungrouped perpendicular distances in the MRDS engine with constant CDS parameters. The best‐fitted model was selected according to Akaike's Information Criterion (AIC) and using chi‐square goodness test. Observation differences among habitat, season, and species were performed using two‐way ANOVA. Type III sum of squares was used to overcome unequal sample size designs. The Poisson regression model, which is recommended for analyzing discrete distributions (count data), was used (Tutz, 2011). Poisson regression model is a Generalized Linear Model (GLM) that is used to model count data and contingency tables. In this model, transect distances, which vary in length, were used to find the effective strip width as shown in Equation 3. This approach was employed to analyze the deviance table (ANOVA). We fitted quasi‐Poisson regression to reduce overdispersion. The optimal Poisson regression model was identified using a stepwise selection process, which simplified the predictor variables in the full model (James et al., 2022).

(3) $$n=e^{\alpha +\beta_{1}\cdot \text{species}+\beta_{2}\cdot \text{season}+\beta_{3}\cdot \text{habitat}+\log\left(\text{Transect distances}\right)}$$

where α represents the intercept, β stands for the coefficient, and ‘*n*’ denotes the number of observations. The factors being considered are habitat type, season, and species.

Tukey's HSD was used for multiple comparisons, multiple comparisons between sex and age groups.

## 3 RESULTS

### 3.1 Model selection

We observed a total of 173 (67, 106) beisa oryx (*Oryx beisa*, dry season *n* = 67, wet season *n* = 106) and 621 gerenuk (*Litocranus walleri*, dry season *n* = 272, wet season *n* = 349) (Table 1). The analysis meets the 60–80 minimum observation standards in line transact sampling. The minimum number of observations was 67 individuals of oryx in the dry season (Table 1).

**TABLE 1:** Population size of gerenuk and beisa oryx recorded per season and habitat type.

Habitat	Covered area (km^2^)	Number observation (*N*)			
		Gerenuk		Beisa oryx	
		Dry	Wet	Dry	Wet
Bushland	20.4	71	80	13	4
Grassland	9.86	52	66	34	77
Wooded grassland	30.78	87	104	20	25
Woodland	38.4	62	99	0	0
Total	99.44	272	349	67	106

The maximum observations were 349 individuals of gerenuk in the wet season. The lowest AIC, ∆AIC (close to zero), and chi‐square tests (*p*‐value > .05) showed that the hazard‐rate key function with an unequal interval group model was selected (Table 2). *p*‐value for all species under two seasons was fitted (*p*‐value > .05), and the model was selected. The *p*‐values for gerenuk and beisa oryx were (.07 and .09) in the dry season and (.17 and .1) in the wet season, respectively. The minimum detection probability of oryx was (*p*_â_ = 76 ± 26) in the wet season and the minimum detection probability of gerenuk was (*p*_â_ = 75 ± 1) during both seasons (Table 2).

**TABLE 2:** Model selection for species survey.

Species	Model	Dry season				Wet season				
		*p*_â_	SE *p*_â_	∆AIC	AIC	*p*‐V	*p*_â_	SE *p*_â_	∆AIC	AIC
Gerenuk	Hn ungroup	.92	.13	6.68	1033	.00	.93	.12	14.27	1274
	Hn grouped	.75	.1	16.0	496	0	.75	.11	0	84
	Hr ungroup	.92	.03	0	1027	.00	.88	.02	0	1260
	Hz grouped	.83	.04	0	480	.17	.83	.03	0	80
Beisa‐oryx	Hn ungroup	.80	.29	2.15	182	.08	.76	.26	4.61	171
	Hn grouped	.82	.33	2.82	82	.04	.81	.27	0	81
	Hr ungroup	.78	.12	0	180	.08	.80	.005	0	167
	Hz grouped	.77	.14	0	80	.10	.82	.13	0	80

Abbreviations: Hn, half‐normal key function; Hr, hazard‐rate key function; *p*_â_, probability of detection; *p*‐V, the *p*‐value for the chi‐square test; SE *p*_â_, standard error for detection probability.

### 3.2 Density and abundance

The coefficient variation (CV%) of the estimated gerenuk density and abundance was less than 40%, which indicates few observations, whereas the coefficient variation (CV%) of estimated beisa oryx was greater than 50%, except one habitat type (woodland, 0%) (Table 3).

**TABLE 3:** Abundance and density estimates of species per season and habitat types.

Species	Habitat	Dry season				Wet season			
		*N*	*N* SE	$\hat{D}$	CV	*N*	*N* SE	$\hat{D}$	CV
Beisa oryx	Bushland	122	95	0.83 ± 0.65	0.77	35	35	0.24 ± 0.24	1.00
	Grassland	301	174	4.60 ± 2.65	0.57	635	302	9.71 ± 4.61	0.47
	Wooded grassland	197	111	0.85 ± 0.48	0.55	230	129	1.00 ± 0.55	0.56
	Woodland	0	0	0.00 ± 0.00	0.00	0	0	0.00 ± 0.00	0.00
	Total	618	245	0.85 ± 0.34	0.38	901	345	1.24 ± 0.47	0.38
Gerenuk	Bushland	737	239	5.03 ± 1.63	0.32	803	233	5.48 ± 1.59	0.29
	Grassland	498	189	7.60 ± 2.90	0.38	653	243	10.0 ± 3.70	0.37
	Wooded grassland	912	229	3.92 ± 1.0	0.25	1098	289	4.72 ± 1.24	0.26
	Woodland	630	166	2.24 ± 0.60	0.26	993	233	3.53 ± 0.83	0.23
	Total	2776	434	3.82 ± 0.60	0.15	3546	511	4.88 ± 0.70	0.14

Abbreviations: CV, coefficient of variation; *N*, estimate of number of mammals in specified area; N.B, estimate of density mammals (number/km^2^); SE, standard error.

The estimated overall total population size and density of each species per km in each habitat type of the two mammal species in the GNP during each season is shown in Table 3. A total of 2776 individuals of gerenuk and 618 individuals of beisa oryx were estimated to live in GNP (Table 3). Estimated density of beisa oryx was (*D* = 0.83 ± 0.65 and 0.24 ± 0.24) in bushland, (*D* = 4.60 ± 2.65 and *D* = 9.71 ± 4.61) in grassland, and (*D* = 0.85 ± 0.48 and *D* = 1.0 ± 0.55) in wooded grassland during dry and wet seasons, respectively (Table 3). Estimated density of gerenuk was (*D* = 5.03 ± 1.63, 5.48 ± 1.59) in bushland (7.60 ± 2.90 and 10.0 ± 3.70) in grassland habitat (3.92 ± 1.0 and 4.72 ± 1.24) in wooded grassland and (2.24 ± 0.60 and 3.53 ± 0.83) in woodland during dry and wet seasons, respectively (Table 3).

### 3.3 Species observation in terms of habitat and season

Species, habitat types, and seasonal variations were computed using the significant interaction/association and non‐significant interaction effects of the Poisson regression model. Habitat versus species (χ^2^ = 364.94, df = 9, *p*‐value < .001) and habitat versus season (χ^2^ = 8.37, df = 3, *p*‐value = .039) showed a significant interaction effect. Species versus season (χ^2^ = 6.80, df = 3, *p*‐value = .07) showed an insignificant interaction effect. For the non‐interaction effect of the Poisson regression model, the dispersion was 1.704 and the dispersion parameter for the Poisson family was taken to be 1. The model overdispersion was adjusted using a quasi‐Poisson regression model, and the dispersion parameter for the quasi‐Poisson family was taken to be 1.704. Species (χ^2^ = 14.3, df = 3, *p*‐value = .0024), Habitat (χ^2^ = 218.04, df = 3, *p*‐value < 0.0001), and season (χ^2^ = 6.31, df = 1, *p*‐value = .012) show significant differences in the non‐interaction effect. The wet season showed a higher record of observation than the dry season (*p*‐value < .0001). Gerenuk showed a higher record than oryx (*p*‐value < .0001). Other contrasts show insignificance non‐interaction effect. In habitat comparison, there were a higher number of observations in the grassland than in the other three habitats (*p*‐value < .0001). Bushland and wooded grassland have insignificant records (*p*‐value = .3418); however, these two habitats showed a higher record than woodland habitat (*p*‐value < .0001).

For pair‐wise multiple comparisons, there was a higher observation in grassland habitat during the wet season than in the dry season (*p*‐value < .001). The highest observation of beisa oryx was recorded in the grassland habitat (*p*‐value < .001). Gerenuk has higher observation records than oryx in bushland habitat and wooded grassland (*p*‐value < .001). However, we observed higher number of individuals of oryx than gerenuk in grassland than woodland habitats. We observed fewer beisa oryx individuals in bushland habitat than in grassland habitat (*p*‐value < .001). Compared to gerenuk, we observed fewer beisa oryx in grassland than in wooded grassland. Gerenuk showed a higher record in bushland, wooded grassland, and woodland habitat.

The main effect model selected, as the dispersion parameter perfectly fitted to 1.0, using estimated marginal means (least‐squares means) of the Tukey's multiple comparison tests. Tukey's multiple comparison tests between juvenile versus sub‐adult male showed insignificance (*p*‐value = .122), while others showed significance differences (*p*‐value = 0). The main effect model was selected, and the dispersion parameter perfectly fitted to 1.0. The Tukey's multiple comparison tests for all sexes (female, male, unknown) showed significant differences (*p*‐value = 0).

**FIGURE 3:**
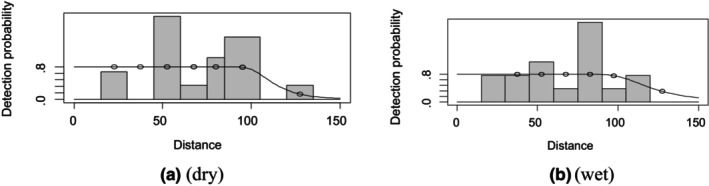
Oryx detection function plot during dry and wet seasons.

For both seasons, all observations beyond the 150 m perpendicular distance were discarded, which is the right truncation. For oryx in the dry season, a manual adjustment of 10 interval groups is needed for 0 to 150 perpendicular distance ranges. The graph shows excessive detections at 45 to 60 and 85 to 105 m transect distances. The graph shows that there was less detection at the beginning of the transect, 30 to 45 and 105 to 120 m distances (Figure 3a). For the wet season, the graph shows that there was no detection at 0 to 15 m perpendicular distance, less detection at 60 to 75 and 90 m to 105 m, and maximum detection at 75 to 90 m. The other interval shows expected detection, which is close to the detection function cure (Figure 3b).

**FIGURE 4:**
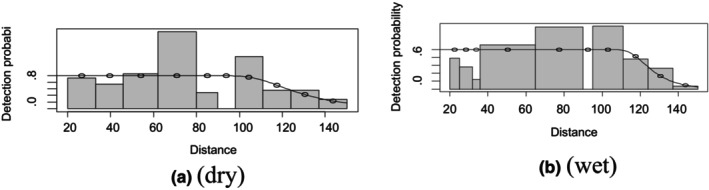
Gerenuk detection function plot during dry and wet seasons.

For gerenuk in the dry season, the plot indicated more detection than expected at the interval 62 to 80 m, and less observation than expected at the interval 80–98 m perpendicular distance; other interval categories show a closer fit to the detection function curve (Figure 4a). For the dry season, there were fewer detections in the first 40 and 90 to 95 m distance intervals than expected in the wet season (Figure 4b). The model fits satisfactory results based on the chi‐square *p*‐value and the lowest AIC value during both seasons (Figure 4).

### 3.4 Population structure

#### 3.4.1 Age and sex structure

**FIGURE 5:**
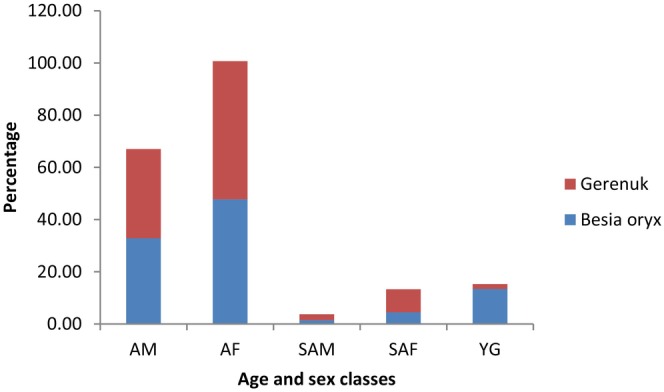
Age and sex percent proportion of populations of beisa oryx and gerenuk during the dry season (AF, adult female; AM, adult male; SAF, sub‐adult female; SAM, sub‐adult male; YG, young).

Adult females comprised the highest proportions for both species during both dry and wet seasons. During the dry season, adult females comprised the highest percent proportion for both gerenuk (52.94%) and beisa oryx (47.76%) (Figure 5, Table 4), whereas sub‐adult males comprised the least percent proportion for both oryx (1.49%) and gerenuk (2.21%) populations during the wet season (0.57%) (Figure 5, Table 4).

**TABLE 4:** Sex and age ratio of gerenuk and beisa oryx in both seasons.

Species	Season	Sex and age categories					Ratio		
							Sex and age		
		AM	AF	SAM	SAF	YG	AM:AF	AF:YG	AM:YG
Beisa oryx	Wet	34	47	0	8	17	1:1.4	1:0.4	1:0.7
	Dry	22	32	1	3	9	1:1.6	1:0.3	1:0.86
	Ava.	27	40	0.5	5	13	1:1.5	1:0.3	1:0.7
Gerenuk	Wet	105	168	16	40	20	1:1.6	1:0.1	1:0.2
	Dry	93	144	6	24	5	1:1.5	1:0.04	1:0.05
	Ava.	99	156	11	32	12	1:1.6	1:0.08	1:0.13

Abbreviations: AF, adult female; AM, adult male; SAF, sub‐adult female; SAM, sub‐adult male; YG, young (Juveniles and Calves).

**FIGURE 6:**
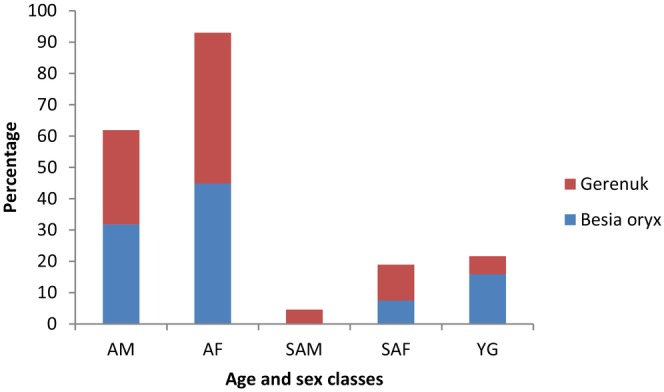
Age and sex percent proportion of populations of beisa oryx and gerenuk during the wet season (AF, adult female; AM, adult male; SAF, sub‐adult female; SAM, sub‐adult male; YG, young).

Likewise, during the wet seasons, adult females comprised the highest percent proportion for both beisa oryx (44.86%) and gerenuk (48.14%). There were no sub‐adult male individuals of beisa oryx recorded during the wet season (Figure 6, Table 4).

#### 3.4.2 Sex ratio

The adult male‐to‐adult female sex ratio (AM:AF) for gerenuk was 1.0:1.6 in the wet and 1:1.5 during the dry season. The adult male‐to‐adult female sex ratio (AM:AF) for beisa oryx was 1.0:1.4 in wet and 1:1.6 in the dry season. The sex ratios of both species were skewed toward females.

## 4 DISCUSSION

### 4.1 Estimation of density and abundance

Ecological studies have shown that variations in abundance and density, or the use of sites or habitats by ungulates, are linked to season, human activity, and the availability of resources (Di Bitetti et al., 2008; Fetene et al., 2019; James et al., 2022; Pérez‐Cortez et al., 2012; Pérez‐Irineo & Santos‐Moreno, 2016; Reyna‐Hurtado et al., 2012). More populations were recorded during the wet season than in the dry season. The most plausible reason for this might be increased human activity, and grazing during the dry season could cause a shortage of available food for herbivores. Furthermore, due to the arid nature of GNP, limited rainfall during the dry season could significantly limit food and cover availability for the ungulate species, which could force populations of the two species to temporarily migrate from the area, leading to poor encounters between the two species in the area (Gandiwa, 2014; Kebede, 2013). Rainfall is one of the important factors that determine the population dynamics of species, especially in African savannah, due to changes in vegetation structure and composition due to limited rainfall (Ogutu et al., 2008; Synodinos et al., 2017). Reproduction, survival, and movements of wild ungulates are highly responsive to rainfall fluctuations, leading to population fluctuations between seasons and vegetation, leading to further desertification and posing negative impacts on ecosystems (Gedir et al., 2023; Omar & Roy, 2010). The study is supported by Mengesha and Bekele (2008) large mammals study conducted in Alitash National Park, northern Ethiopia, which stated that the abundance of species is affected by the availability of food and cover, which is influenced mainly by vegetation composition and structure. Drought has had an important effect on herbivores in savannah species living in the Mara Serengeti ecosystem, which have declined by 58% in the last 20 years due to drought‐related effects on vegetation (Ottichilo et al., 2000). More mammals were observed in the core area, particularly in the Dodo and Elbenki plains of the park, where higher individuals of beisa oryx were recorded during the wet season.

Furthermore, various studies have reported that livestock encroachment into wildlife habitats has remained a serious challenge for conservation of wildlife species, causing a decline in populations of wild animals (Gebremedhn et al., 2023; Girma et al., 2018; Macura et al., 2011; Stephens et al., 2001). Several wildlife surveys have also reported that livestock encroachments and human settlements reduced the detection probabilities of wild animals (Dinakaran & Anbalagan, 2007; Milner et al., 2007; M'soka et al., 2017; Stankowich, 2008).

Geralle National Park harbors a comparable population estimate of beisa oryx (618) as compared to other protected areas in Ethiopia, such as Awash National Park (405, Enawgaw, 2004) and Alledeghi Wildlife Reserve (now upgraded to a national park) (1119, Simeneh et al., 2016). In this regard, the study discovered a healthy population of the species that can be translated into conservation measures. Populations vary between seasons in GNP; a higher number of individuals were recorded during the wet season than during the dry season. There might be a number of reasons for such a seasonal difference. The variation observed might be caused by seasonal changes in the resource requirements of the species in different habitat types. Drought causes the animals to shift to less productive and more drought‐tolerant plant species (Batbaatar et al., 2022; Mengesha & Bekele, 2008). The availability of resources during the wet season probably caused the beisa oryx to aggregate in large numbers, spend more time feeding in grassland and wooded grassland habitats compared to others in GNP as a result of its narrow range of tolerances to environmental conditions and human activities, and spend less time in bushland and woodland habitat types. The difference in density estimate among habitat types could be because of variation in habitat selection characteristics and protection, as stated by Linnell et al. (2015) and Wegge et al. (2009). Smith et al. (2022) also stated that density‐dependent habitat factors drive changes in the abundance of elk in Yellow Stone National Park. It is evident that resources are less available during the dry season compared to the wet season; thus, such resource scarcity during the dry season might have caused some populations of oryx to immigrate to other surrounding areas in search of better sources of forage. Following rains in the wet season, they mostly return to grassland habitat to feed on fresh, nutritious grass shoots. In the wet season, the Dodo and Elbenki plains were very green, and there were more oryx in the plain (Ottichilo et al., 2000). However, when there is no rain, the majority of beisa oryx leave the grassland and might move to bush and wooded grassland. In the dry season, as the grass gradually loses its moisture and dries up, it moves for alternative food sources and shade (Gedir et al., 2023). The beisa oryx population was more abundant in grassland habitat during the wet season, while there were more populations in wooded grassland and bushland habitat during the dry season. This might be due to the availability of sufficient food and better vigilance for predators during the dry season in grassland habitat (Admasu et al., 2016; Atkinson et al., 2022; Enawgaw, 2004).

The gerenuk population density recorded (3.82 ± 0.60) indicates a healthy population and the crucial role of GNP for the conservation of the species. It was reported that density estimates of gerenuk range from a maximum of 0.2/km^2^ on aerial surveys to 1.0/km^2^ from road counts and up to 8.0/km^2^ in particularly favorable habitats, such as Samburu G.R. in Kenya (Leuthold, 2013). The highest gerenuk densities were recorded in grassland and bushland habitats during both dry and wet seasons. This was due to the better quality of fodder available during the wet than dry season in the grassland habitat, because of the availability of growing grasses and bushes during the rainy season, which provides better nutritional requirements for the species, similar studies by Kasiringua et al. (2019) in Namibia and (Okello et al., 2016) in Kenya witnessed this. Furthermore, the relatively open nature of grassland and bushland could provide a better opportunity for vigilance for the species (Tamrat et al., 2020). It was reported that gerenuk occurs from open treeless plains of Serengeti to less dense bushlands but is known to avoid tick woodlands (Bärmann et al., 2021; IUCN, 2024; Leuthold, 2013). The availability of food during the wet season probably made gerenuk has a larger population size than in the dry season and spend more time feeding on grassland and bushland habitats than woodland habitat. Gerenuk was well observed across all habitat types. Gerenuk had a wide habitat range as compared to oryx. The reason might be that span style = “font‐family: ‘Times New Roman’; letter‐spacing: −0.05pt” > gerenuk is the most abundant species in GNP, with a wide range of distribution/habitat and a large population. Gerenuk density and abundance were higher in the wet season than in the dry season. Similar studies by Admasu et al. (2016) in Haledeghe Wildlife Reserve (HWR) and (Okello et al., 2016) in Kenya‐Tanzania borderland reported higher gerenuk density during the wet season than the dry season.

### 4.2 Population structure

The knowledge of the sex ratio and age distribution of individual mammals is vital for evaluating the viability of a species because these variables reflect the structure and dynamics of the population (Butka & Freedberg, 2019; Mamo et al., 2010; Wilson et al., 1996). Understanding sex and age population structure is important for evaluating the viability of the species, as these variables reflect the structure and dynamics of a species population (Abate & Girma, 2023; Groves, 2005). The sex ratio for both species was biased or skewed toward females. This might be due to the probability of an increase in the killing of males due to hunting and predation. In most African ungulate populations, males sometimes leave the herd and move to less favorable habitats and could suffer increased predation pressure compared with females of the same age classes (Tsegaye et al., 2015). In a study by Kasiringua et al. (2019), on ungulate populations in Zambia male, female sex ratios were biased toward females, and seasonal variation between sex and age has also been observed in the population. Ungulate species that leave open habitats leave their young in bushland or forests to hide them from potential predators (McKay & Finnegan, 2023). Okello et al. (2016) explained that a possible reason for an unequal sex ratio and a lower proportion of adult males in most of the species might be related to poaching pressure, in which the adult males are mostly selected by poachers, predation pressure on males (both by natural predators and humans), or emigration of males to other habitats for food.

Male‐biased hunting practices of the species by local communities might contribute to the lower number of adult males (Mamo et al., 2010). During the dry season, there was a lack of water and may have been forced to cross the bush vegetation cover in search of water. In general, unequal sex ratios within a given species can occur, favoring either males or females, and are explained as a function of species‐specific reproductive, foraging, and defensive behavioral traits (Admasu et al., 2016; Mengesha & Bekele, 2008). Similar results were reported in ungulate population studies by Enawgaw (2004) in Awash National Park (ANP) and Admasu et al. (2016) in Hallidige Wildlife Reserve (HWR). Competition among males in African ungulates could also force the bachelor males to migrate to less suitable habitats that are poor in food quality, exposing them to predators and hunters (Alemu et al., 2016). Some studies also suggested that male‐biased hunting pressure limits wildlife populations because female fecundity may be reduced when males are selectively removed, resulting in reproductive collapse in the antelope population (Mamo et al., 2010).

More records of juveniles were observed during the wet season, as they mainly give birth during the wet season. The difference in the number of young between dry and wet seasons might be due to birth of juveniles by females during the wet season, as noted by scholars (Mamo et al., 2010; Okello et al., 2016). The birth rate peaks during the wet season due to more abundant forage resources during the wet season as compared to the dry season (Ogutu et al., 2015). Furthermore, young usually hide inside dense and tall grasses and in the bushes during the dry season, until they are strong enough to run fast and escape from predators, which reduces their sightings during the dry season. This is also supported by the findings of Enawgaw (2004), which confirmed that ungulates such as beisa oryx at the early stages of their lives are highly vulnerable to predators. A study by Asefa et al. (2017) found that GNP is home to top predators such as lions, common jackals, spotted hyenas, leopards, and cheetahs that support the claim. The irregular availability of water and rain affects the age and sex structure of herbivores during the wet and dry seasons, juveniles, and older individuals and males might have a lower survival rate than females, as stated by Kasiringua et al. (2019). Dry seasons and droughts might cause more mortality than other time and lead to age–sex‐dependent mortality, where most young and old are killed (Okello et al., 2016).

## 5 CONCLUSION AND RECOMMENDATIONS

It can be concluded from the results of the study that GNP is home to previously undiscovered viable populations of the endangered beisa oryx and near‐threatened gerenuk. This signifies the crucial role of the national park in the conservation of the species. Season that determines rainfall availability and consequent availability of forage and cover has a significant impact on the abundance of the two species. Habitat vegetation characteristics such as availability of grassland and less dense forest favored populations of both beisa oryx and gerenuk. The population structure was skewed toward females. Generally, relatively higher proportion of adult females observed indicates a merely healthy population, assuming that there is greater opportunity for increasing individuals in the population through new births. The reasonably good populations of young for both species also indicate growing populations. So it is recommended to undergo in‐depth population studies, including other species available in the national park and their habitat components, so as to design sound, sustainable conservation measures for the wildlife resources in the area.

## AUTHOR CONTRIBUTIONS

**Melkamu Aychew:** Data curation (lead); investigation (equal); methodology (supporting); writing – original draft (supporting). **Zerihun Girma:** Conceptualization (lead); formal analysis (equal); investigation (equal); methodology (equal); supervision (lead); writing – original draft (equal); writing – review and editing (lead).

## FUNDING INFORMATION

The research was conducted with financial support from African Wildlife Foundation through Ethiopian Wildlife Conservation Authority.

## CONFLICT OF INTEREST STATEMENT

We the authors declare no competing interests.

## Supporting information


Data S1:

## Data Availability

The data that support the findings of this study are uploaded as supporting document.
